# Integrated tumor and germline profiling of lynch syndrome in a North Indian cohort

**DOI:** 10.3389/fonc.2026.1804614

**Published:** 2026-05-19

**Authors:** Himanshi Diwan, Anurag Mehta, Saudamini Sharma, Sakshi Mattoo, Shalini Agnihotri

**Affiliations:** 1 Department of Laboratory Services and Molecular Diagnostics, Rajiv Gandhi Cancer Institute and Research Centre (RGCIRC), Delhi, India; 2 Department of Molecular Diagnostics, Rajiv Gandhi Cancer Institute and Research Centre (RGCIRC), Delhi, India; 3 Department of Research, Rajiv Gandhi Cancer Institute and Research Centre (RGCIRC), Delhi, India

**Keywords:** colon cancer, endometrial cancer, founder mutation, inherited, lynch syndrome

## Abstract

**Background:**

Lynch syndrome is the leading hereditary cause of colorectal and endometrial cancers, but data on germline mutations in the Indian population are insufficient. This study assessed patients with Lynch syndrome-related tumors using tumor mismatch repair (MMR) immunohistochemistry and/or microsatellite instability testing, followed by germline analysis of mismatch repair genes from blood samples. The goal was to determine the prevalence and molecular profile of Lynch syndrome in a North Indian population and to identify common pathogenic variants with high frequency in this population.

**Methods:**

We retrospectively evaluated 136 individuals with Lynch syndrome–associated malignancies or a strong personal or family history who were assessed at a tertiary cancer center in North India between January 2020 and February 2025. Tumor MMR status was determined by immunohistochemistry or by surrogate Microsatellite Instability testing. Germline analysis of MLH1, MSH2, MSH6, and PMS2 was performed using next-generation sequencing–based assays.

**Results:**

Lynch syndrome was confirmed in 53 individuals (38.9%), with colorectal cancer representing 71.7% (38 cases) and endometrial cancer accounting for 28.3% (15 cases) cases). *MLH1* was the most frequently mutated gene (53.57%), followed by MSH2, MSH6, and PMS2. A recurrent pathogenic variant, *MLH1* c.306G>T; p.(Glu102Asp), accounted for nearly one-quarter of Lynch syndrome cases and was observed exclusively in individuals of Punjabi ancestry, suggesting a strong founder effect.

**Conclusions:**

Lynch syndrome contributes substantially to colorectal and endometrial cancers in the North Indian population. MLH1 gene alterations predominate, and the presence of a founder variant is reaffirmed. 38.9% of reported cases are caused by LS. Germline testing helps identify probands and FDRs who are healthy mutation carriers. Risk-reduction strategies for them can help reduce the risk of CRC in the Indian population.

## Introduction

Lynch syndrome is a hereditary condition that increases cancer risk and results from germline pathogenic mutations in DNA mismatch repair (MMR) genes such as *MLH1, MSH2, MSH6*, and *PMS2*. Defects in any MMR gene significantly raise the lifetime risk of developing cancers, mainly colorectal and endometrial, but also ovarian, gastric, small intestine, hepatobiliary, urinary tract, brain, and sebaceous gland tumors (1–3).

The origins of Lynch syndrome research trace back to 1895, when Dr. Warthin documented hereditary cancer clustering in a family later known as Family G (4). Subsequently, clinical diagnostic frameworks, such as the Amsterdam and Revised Bethesda criteria, were developed to facilitate recognition of affected families (5–7). In contemporary practice, universal surrogate testing for Lynch syndrome, using loss of MMR proteins or microsatellite instability testing, is the standard of care for colorectal and endometrial cancers (8). Defective mismatch repair leads to length alterations in repetitive DNA sequences (1–6 nucleotide repeats) known as microsatellites. Inactivation of *MLH1*, *MSH2*, or *PMS2* is typically associated with high-level microsatellite instability (MSI-H). In contrast, *MSH6* deficiency predominantly affects mononucleotide repeats and may result in minimal or absent MSI on PCR-based assays.

The relative risk of cancer varies depending on the affected MMR gene. *MLH1* and *MSH2* mutations are associated with the classic Lynch syndrome phenotype, characterized by early-onset colorectal or endometrial cancer and a lifetime cancer risk of approximately 50% (9). In contrast, mutations in *MSH6* and *PMS2* are associated with late-onset tumors and lower overall penetrance, with *MSH6* mutations strongly associated with endometrial carcinoma (10–12). The lower cancer incidence in *PMS2* mutation carriers is thought to reflect partial functional redundancy and a lesser overall impact of *PMS2* loss on mismatch repair fidelity.

Early detection of Lynch syndrome is essential because organized surveillance programs greatly decrease cancer rates and disease-specific deaths among affected individuals and first-degree relatives with the harmful genetic mutation. This study aimed to define the incidence, genetic spectrum, and clinicopathological features of Lynch syndrome in a North Indian cohort and recurrent *MLH1* p.(Glu102Asp) variant, reaffirming that this is a regional founder mutation as described previously in the literature (13, 14).

## Materials and methods

### Research setting and subjects

From January 2020 to February 2025, one hundred thirty-six patients diagnosed with Lynch syndrome-associated cancers and exhibiting a strong family or personal history, deficient mismatch repair (MMR) status on immunohistochemical evaluation, or microsatellite instability on PCR-based MSI testing were included in the study. As this was a retrospective study including cases evaluated over several years, some variability in tumor testing strategies arose from the evolving availability of diagnostic assays at our center. During the earlier part of the study period, mismatch repair (MMR) immunohistochemistry served as the primary screening modality, as MSI-PCR testing was not yet available at our institution. Following the implementation of MSI-PCR testing, a subset of patients underwent both MMR immunohistochemistry and MSI-PCR to increase diagnostic confidence during the initial phase of adoption. Subsequently, MSI-PCR was primarily used to screen for colorectal tumors, whereas MMR immunohistochemistry remained the primary screening method for endometrial tumors. In cases of endometrial cancer, MSI-PCR was performed in selected situations where additional validation of mismatch repair status was required. This reflects the dataset’s retrospective nature and the gradual evolution of institutional diagnostic practices during the study period.

Each eligible participant was thoroughly informed and counseled by the institutional genetic counselor regarding all aspects of germline testing for Lynch syndrome. Written informed consent was obtained for both germline testing and the use of their information for research purposes. Participants were screened for mutations in the DNA mismatch repair genes (*MLH1, MSH2, MSH6, and PMS2*) using next-generation sequencing (NGS).

A total of 136 individuals underwent germline Lynch syndrome testing, comprising 132 probands (index cases) and 4 unaffected first-degree relatives (FDRs) of deceased cancer patients.

### Isolation of DNA from blood, NGS, and data analysis

Genomic DNA was isolated from 2 mL of peripheral blood from the index case using the commercially available Qiagen DNeasy Blood and Tissue kit (Qiagen NV, Hilden, Germany) following the manufacturer’s instructions. The isolated DNA was quantified using the Qubit 3.0 fluorometric quantitation system (Thermo Fisher Scientific, Waltham, MA, USA). The NGS library was prepared manually from 10 ng of isolated DNA using a customized in-house 4-gene panel that interrogates single-nucleotide variants, multinucleotide variants, and indels in the *MLH1, PMS2, MSH2*, and *MSH6* genes. This study did not evaluate copy number variations (CNVs) or large genomic rearrangements, including EPCAM deletions, as part of the analysis. Data from the runs were evaluated for quality metrics, such as the number of mapped reads, average base coverage depth, coverage uniformity, and coverage at 1 ×, 30 ×, and 100 ×, along with strand bias, using thresholds defined in the National Cancer Institute-Molecular Analysis for Therapy Choice (NCI-MATCH) trial (15). Variants were classified based on the guidelines provided by the American Society of Human Genetics and the American College of Medical Genetics and Genomics for standardizing the interpretation and reporting of sequence variants (16).

*KRAS, NRAS*, and *BRAF* mutation analysis was performed using the Entrogen real-time polymerase chain reaction kit, and MLH1 promoter hypermethylation testing was performed by pyrosequencing with Qiagen kits.

### Statistical analysis

Descriptive statistics were used to summarize the data. The data were analyzed statistically using SPSS version. 23.0 (IBM Corp., Armonk, NY, USA). The statistical analysis consisted of calculating means and proportions. Appropriate tests of significance were applied, and a p-value of <.05 was considered significant.

## Results

**Figure 1:**
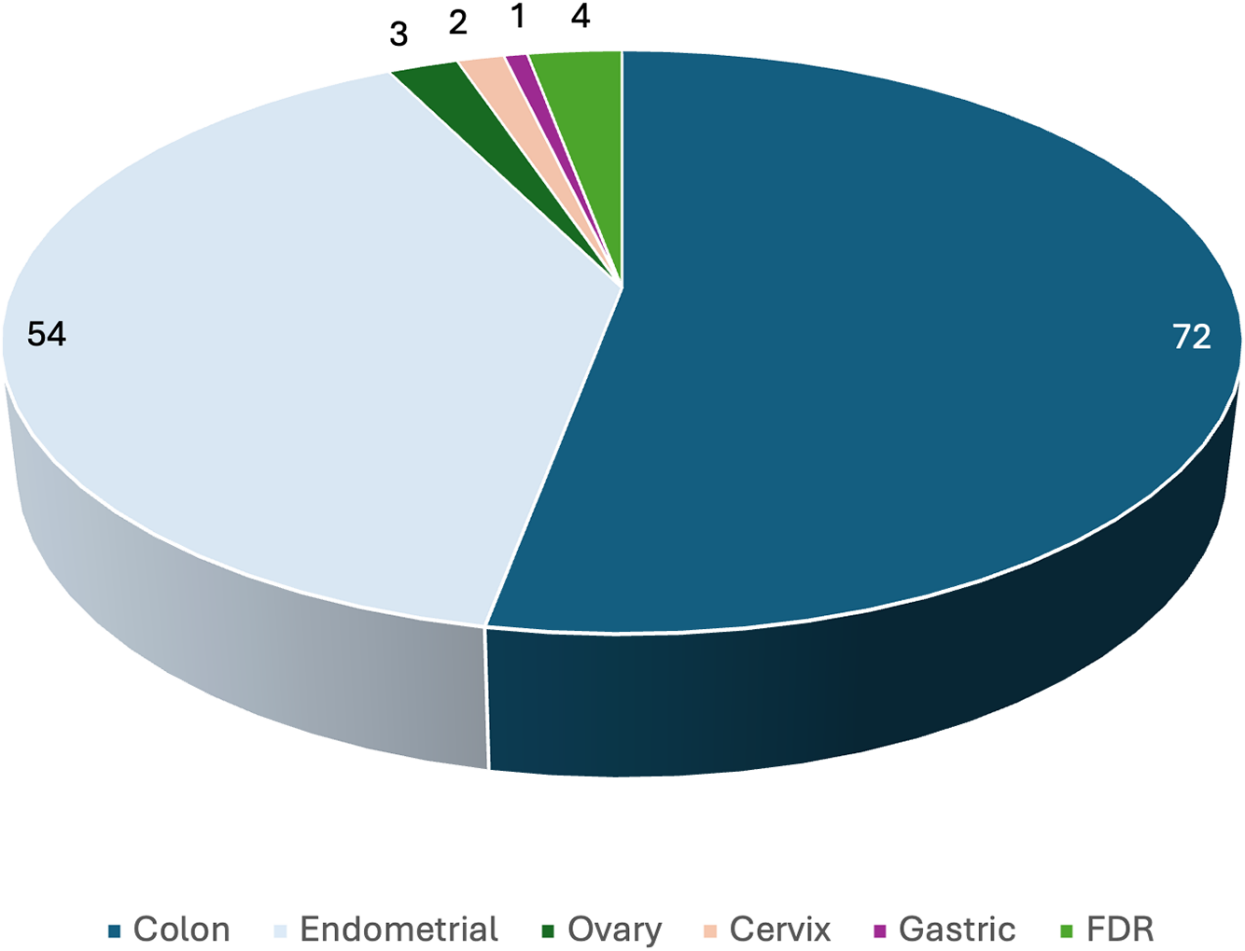
Distribution of cancer types across the studied cases.

This is a retrospective study from a tertiary care cancer institute, which included patients with Lynch syndrome-associated cancers with a strong family or personal history or deficient MMR or microsatellite instability from January 2020 to February 2025. A total of 136 patients underwent germline Lynch testing, of which 72 cases presented with colon carcinoma, 54 with endometrial carcinoma, 3 with ovarian carcinoma, 2 with cervical carcinoma, and one with gastric carcinoma. Of them, 15 patients had a personal history of multiple cancers (**Table 1**). Four healthy first degree relatives (FDRs) of the deceased cancer patients underwent germline testing owing to their strong family history (**Figure 1**). Of these 4 healthy FDRs, only one case was found to harbor a deleterious germline mutation, i.e., *MLH1* Reference Genome Build GRCh37 (NM_000249.4): c.306G>T, p.(Glu102Asp) variant, confirming Lynch syndrome.

**Table 1:** Clinical details of cases with a history of multiple cancers in the past.

Cases with multiple cancers	Cancer type	Mutation in the MMR gene
Case 1	Sigmoid +Jejunum (Metachronous)	Yes (*MLH1*)
Case 2	Rectum +Ascending colon + Bladder (Metachronous)	Yes (*MSH2*)
Case 3	Rectosigmoid + Transverse colon + Small Intestine (Metachronous)	Yes (*MLH1*)
Case 4	Ascending colon, Transverse colon, Cervix (Metachronous)	Yes (*MLH1*)
Case 5	Endometrial + Papillary thyroid carcinoma (Metachronous)	Yes (*MSH2*)
Case 6	Endometrial+ Ovary (Synchronous)+ Colon + Rectum (Metachronous)	Yes (*MLH1*)
Case 7	Colon+ Esophagus+ Rectosigmoid (Metachronous)	No
Case 8	Anorectum Sigmoid + Descending colon (Metachronous)	No
Case 9	Ascending colon + Prostate (Metachronous)	No
Case 10	Endometrial + Kidney (Metachronous)	No
Case 11	Endometrial + Breast (Synchronous)	No
Case 12	Endometrial + Ovary (Synchronous)	No
Case 13	Endometrial + Ovary(Metachronous)	No
Case 14	Cervix + Papillary thyroid carcinoma (Metachronous)	No
Case 15	Colon + Prostate (Metachronous)	No

Of the 132 carcinoma cases, MMR immunohistochemistry (IHC) results were available for 102. MSI-PCR results were available for 44 cases, including 21 that also underwent MMR-IHC and 23 that underwent MSI-PCR alone. Overall, 21 cases had both MMR-IHC and MSI-PCR results, while 7 cases had neither test performed nor had insufficient tissue for analysis.

A total of 72 cases of colon carcinoma (22 females and 50 males) were included in the study. These included 24 left-sided tumors, 45 right-sided tumors, and one case with tumors on both sides. Two patients were referred solely for germline Lynch syndrome testing, and no electronic medical records were available for them. The average age of the cohort was 50.22 years.

**Figure 2:**
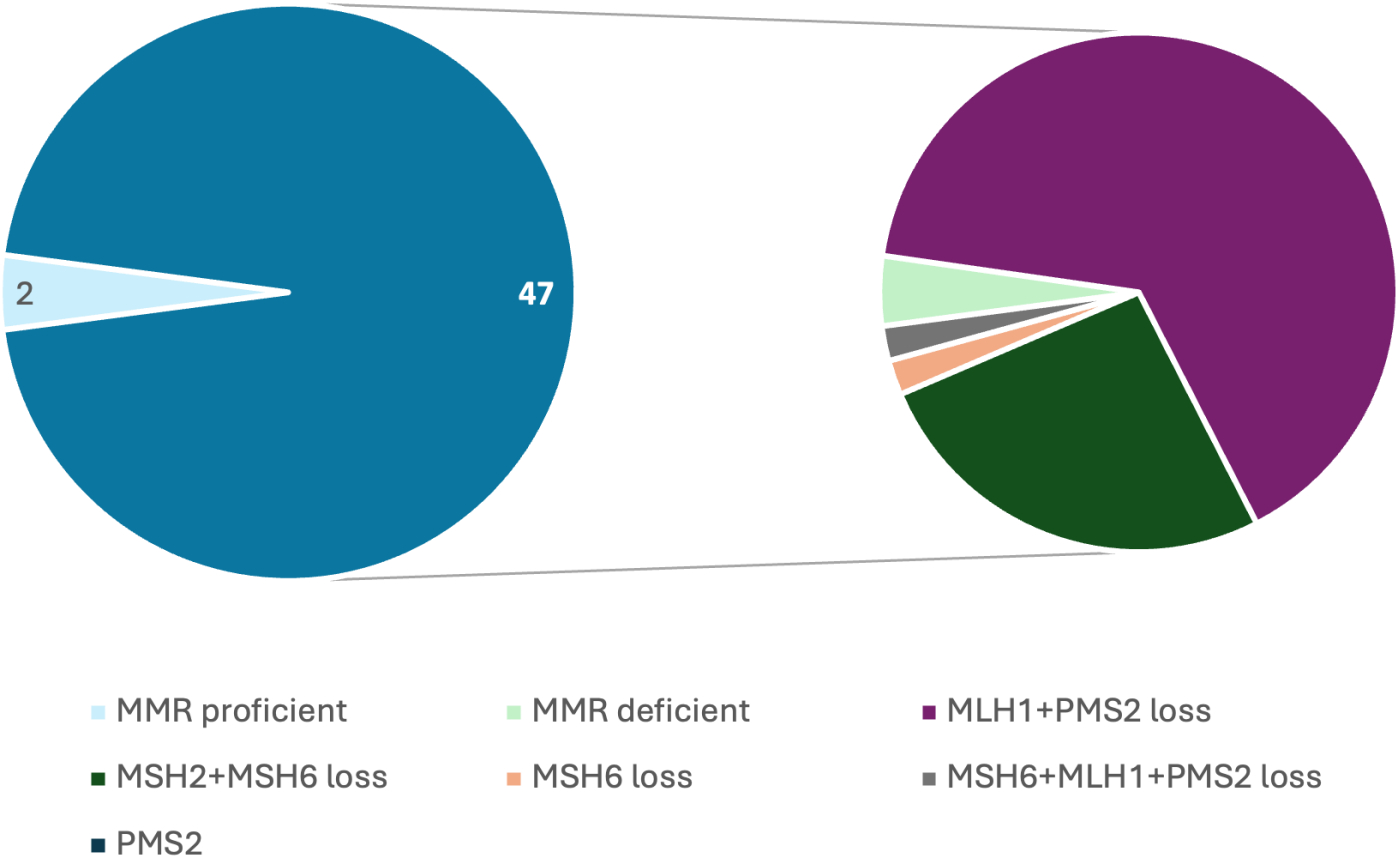
Frequency of MMR protein loss on IHC across colon carcinoma cases included in the study.

As this was a retrospective study, MMR testing was used as the primary screening modality initially due to the nonavailability of MSI-PCR at our center. 49 underwent MMR testing by IHC (**Figure 2**), and MSI-PCR results were available for the remaining 22 cases. MMR status could not be obtained for one case. Of the 49 cases evaluated by immunohistochemistry (IHC), 47 were identified as MMR-deficient. Of the 22 cases tested by MSI-PCR, 19 demonstrated microsatellite instability.

Both MMR protein expression by IHC and MSI-PCR testing were available in 19 cases. Concordant results between MMR IHC and MSI status were observed in 18 cases (94.7%). One case demonstrated an isolated loss of MSH6 expression on IHC while remaining microsatellite-stable on MSI analysis.

Loss of MLH1 and PMS2 expression was observed in 30 of the 47 MMR-deficient cases. *BRAF* mutation analysis data by real-time PCR using the Entrogen kit were available in 23 cases, of which 4 (17.4%) harbored the *BRAF* p.(Val600Glu) mutation. Of these 4 cases, 1 was associated with germline-confirmed Lynch syndrome. *MLH1* promoter methylation analysis was performed in 10 cases, of which 4 were methylated. None of the *MLH1*-hypermethylated cases were associated with germline-confirmed Lynch syndrome. *KRAS* mutation analysis by real-time PCR using the Entrogen kit was available in 9 cases, with mutations identified in 3 cases (33.3%). Of these, 1 case was associated with Lynch syndrome.

Twelve cases showed combined loss of MSH2 and MSH6, three had isolated loss of MSH6, one case showed loss of MSH6, MLH1, and PMS2, and one case demonstrated isolated PMS2 loss.

A total of 29 of the 47 MMR-deficient cases (61.7%) were subsequently found to harbor a mutation in one of the tested MMR genes (**Table 2**).

**Table 2:** Frequency distribution of mutations in d-MMR cases in colon carcinoma.

Cases with d-MMR (Total=47)	Number of cases with germline mutation
MLH1+PMS 2 loss (n=30)	19
MSH2+MSH6 loss (n=12)	7
MSH6 loss (n=3)	1
MSH6+MLH1+PMS2 loss (n=1)	1
PMS2 loss (n=1)	1

Eleven cases demonstrated concurrent loss of MLH1 and PMS2 expression on immunohistochemistry (IHC) but did not harbor germline mutations in mismatch repair (MMR) genes. Of them, somatic *MLH1* hypermethylation was identified in four cases. Two cases harbored the *BRAF* p.(Val600Glu) mutation, as detected by real-time PCR with the Entrogen kit; one also demonstrated somatic *MLH1* hypermethylation. Additionally, one case showed a *KRAS* exon 2 mutation detected by real-time PCR using the Entrogen kit.

Of the 72 colon carcinoma cases included in the study, 58.33% (n = 42) demonstrated a germline mutation in *MSH2, MSH6, MLH1*, or *PMS2*. Among these 42 individuals, 13 were female, and 29 were male. The youngest patient was 21 years old, and the mean age was 50.22 years (range: 21–78 years).

No statistically significant difference was observed in the prevalence of Lynch syndrome between male and female patients (Fisher’s exact test; p = 0.92). The 95% confidence intervals for prevalence ranged from 38–50% in females and 45–72% in males, reflecting only minimal differences in proportions. Effect size estimates were negligible, with relative risks of 1.02 and odds ratios of 1.05.

**Figure 3:**
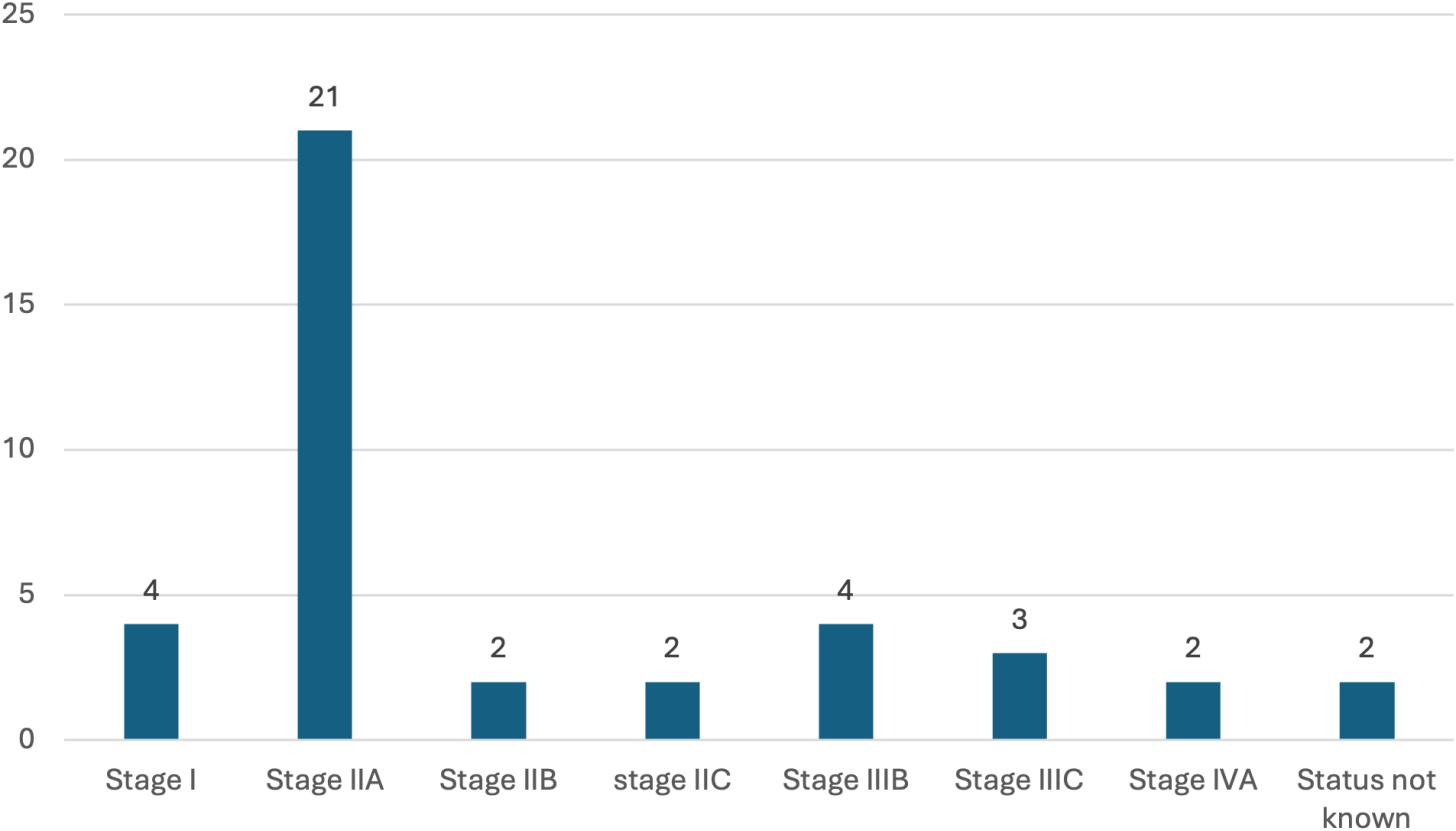
AJCC stage distribution in lynch syndrome-associated colon carcinoma cases in the study.

Of these 42 mutated patients, two patients presented with metastatic disease at diagnosis, and six had nodal metastasis. The majority were classified as Stage II (n = 25/42; 59.52%), including 21 patients with Stage IIA, 2 with Stage IIB, and 2 with Stage IIC disease. The AJCC stage distribution is summarized in **Figure 3**.

Of these 42 mutated patients, a total of 29 cases were identified as MMR-deficient, while MMR testing was not performed in 13 cases. Among these 13 cases, MSI-PCR was carried out in 12, and all demonstrated microsatellite instability. No records were available for one case, as it had been referred solely for germline testing. Twelve cases underwent both MSI-PCR and MMR-IHC testing, and all were found to be MMR-deficient and MSI-high.

An interesting case involved a 72-year-old female with a right-sided colon tumor, staged as pT4bN2 (Stage IIIC), exhibiting a poorly differentiated adenocarcinoma on histopathology. This tumor harbored a *BRAF* V600E mutation, and germline analysis revealed a pathogenic *PMS2* variant [NM_000535.7: chr7:6018263, c.2239A>T, p.(Arg747*), Reference Genome Build GRCh37]. Caution is advised when excluding cases with *BRAF* p.(Val600Glu) for Lynch testing, as in rare instances, both can coexist. Other criteria, including phenotype and family history, should also be considered.

An intriguing case in our cohort involved a patient with multiple primary malignancies, including a rectosigmoid carcinoma diagnosed in 2003, a transverse colon carcinoma in 2021, and a small intestinal tumor in 2022. All tumors demonstrated MSI-high status. Molecular profiling using the EntroGen assay identified a *KRAS* exon 2 mutation, while germline analysis revealed a pathogenic *MLH1* Reference Genome Build GRCh37 (NM_000249.4): c.306G>T, p.(Glu102Asp) variant, confirming Lynch syndrome. This case underscores that *KRAS* mutations can occur in Lynch-associated tumors and do not exclude the diagnosis, especially in the context of confirmed germline MMR gene alterations.

**Figure 4:**
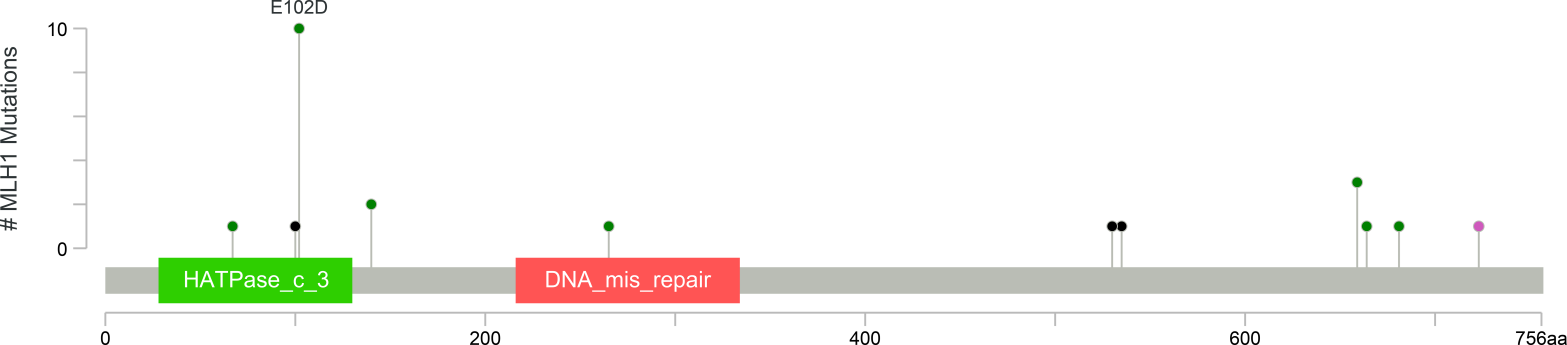
Lolliplot chart demonstrating *MLH1* variants observed in colon carcinoma case cohort (17).

**Figure 5:**
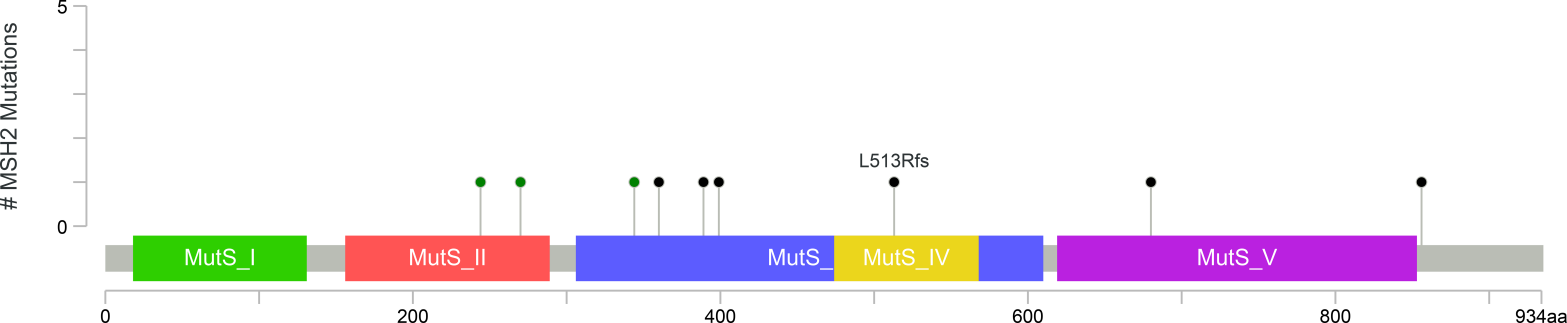
Lolliplot chart demonstrating *MSH2* variants observed in colon carcinoma case cohort (17).

Among the 42 colon carcinoma cases with germline alterations, 38 carried pathogenic variants in MMR genes and classified as Lynch syndrome, while four harbored variants of uncertain significance in *MLH1*, *MSH2*, and *MSH6*, respectively. *MLH1* was the most frequently affected gene (25 cases; 59.5%), followed by *MSH2* (10 cases; 23.8%), and *PMS2* (3 cases; 7.1%) (**Figures 4**, **5**; **Table 3**).

**Table 3:** Deleterious variants in *PMS2* in colon cancer cohort.

Gene	Variant
*PMS2*	(NM_000535.7): c.1275dup; p.(Leu426Serfs*32)
*PMS2*	(NM_000535.7): c.1239delA; p.(Asp414Thrfs*34)
*PMS2*	(NM_000535.7): c.2239A>T; p.(Arg747*)

Of the 25 patients with pathogenic *MLH1* variants, *MLH1* Reference Genome Build GRCh37 (NM_000249.4): c.306G>T, p.(Glu102Asp) was the most prevalent, detected in 10 cases. This variant represented 40% of all *MLH1* mutations and 23.8% of Lynch syndrome-associated colon carcinomas. Interestingly, all carriers were of Punjabi descent, reaffirming its founder effect in this population which has previously been described in the literature (13, 14). Three of these cases also had a strong family history of Lynch syndrome-related cancers, suggesting possible familial aggregation; however, no conclusions regarding penetrance can be drawn in the absence of segregation data. Among these cases, one presented with nodal metastasis. Staging distribution included 1 case of Stage I, 7 cases of Stage IIA, 1 case of Stage IIC, and 1 case of Stage IIIC disease.

Variants of uncertain significance (VUS) were identified in 4 cases diagnosed with colon carcinoma. The details are provided in **Table 4**.

**Table 4:** Variant of uncertain significance identified in colon cancer cohort.

Gene	Variant	ClinVar	ACMG classification
*MSH2*	(NM_000251.3): c.731T>C; p.(Leu244Ser)	VUS	VUS (PP3, PM2, BP1)
*MSH6*	(NM_000179.3): c.2017C>A; p.(Pro673Thr)	Conflicting	VUS (PM2, PP3, BP1)
*MSH6*	(NM_000179.3): c.4065_4066insGACT; p.(Leu1356Aspfs*4)	VUS	VUS (PVS1, PM2, BP6)
*MLH1*	(NM_000249.4): c.1559-11T>C	Conflicting	Likely benign (BP4, BP6, PM2)

Although this frameshift variant in *the MSH6* gene results in premature truncation of the protein, it is still classified as a variant of uncertain significance in ClinVar. The mutation results in a truncated protein due to a frameshift insertion in the last exon, producing a protein that is only four amino acids shorter. It appears that the *MSH6* protein, shortened by just four amino acids, probably retains its full functional capacity. However, no functional assay was performed for this variant due to logistic issues.

A total of 54 endometrial carcinoma cases were included in the study, comprising 17 high-grade and 35 low-grade tumors. Clinical information was unavailable for two instances in the departmental archives. The cohort’s mean age was 58.4 years.

MMR testing was performed in 49 cases, and one case underwent MSI-PCR analysis and no records were available for 4 cases. MLH1 and PMS2 loss was identified in 34 cases, MSH2 and MSH6 loss in 4 cases, isolated MSH6 loss in 10 cases, and isolated PMS2 loss in 1 case (**Table 5**).

**Table 5:** Number of endometrial carcinoma cases with variant in MMR genes and their MMR status.

MMR protein loss	Number of cases with pathogenic/likely pathogenic/VUS variant
MLH1+ PMS2 loss (n=34)	8 (7 pathogenic/Likely pathogenic; 1 VUS)
MSH2+MSH6 loss (n=4)	2
MSH6 loss(n=10)	5
PMS2 loss (n=1)	0
MMR status not known (n=5)	2 (1 Pathogenic; 1 VUS)

In one case, a small biopsy suggested d-MMR, but repeat testing on the surgical resection specimen showed proficient MMR. Due to this discordance, Lynch syndrome testing was performed, which revealed no germline mutations in MMR genes. Another case showed immunohistochemical loss of MLH1 and PMS2, while MSI-PCR demonstrated microsatellite-stable status; subsequent germline testing did not identify any deleterious MMR variants.

Germline testing for Lynch syndrome revealed pathogenic/likely pathogenic variants in 17 cases and variants of uncertain significance (VUS) in 2 cases. Clinically, 9 cases were Stage IA, 5 were Stage IB, 1 was Stage II, and 2 lacked staging information; one patient presented with nodal metastasis. Overall, 15 cases were MMR-deficient, and MMR status was unavailable in two cases. p53 IHC status was available in 11 cases; one grade 1 endometrioid carcinoma demonstrated a p53 mutant phenotype and harbored a VUS variant in the *PMS2* gene.

Among the 34 cases with MLH1 and PMS2 loss, 8 carried variants in MMR genes: 7 pathogenic/likely pathogenic and 1 VUS in PMS2. *MLH1* variants were detected in 5 cases, three of which had the identical variant *MLH1* Reference Genome Build GRCh37 (NM_000249.4): c.306G>T, p.(Glu102Asp). All three patients originated from the northwestern province of India and were predominantly Punjabi. One case demonstrated a frameshift pathogenic variant in MSH6, and another harbored a pathogenic synonymous *PMS2* variant, PMS2 Reference Genome Build GRCh37 (NM_000535.7): c.903G>A; p.(Lys301=). Although silent at the amino acid level, the alteration affects the final base of the exon, part of the 5′ splice site, and is therefore likely to disrupt RNA splicing.

Three cases of endometrioid ovarian carcinoma, two cases of cervical carcinoma (one case also had metachronous papillary thyroid carcinoma), and one gastric carcinoma were also tested for Germline Lynch syndrome; however, none revealed any pathogenic variant in MMR genes.

Four cases underwent screening for Lynch syndrome despite a lack of a strong family history. As the affected members were deceased, the subjects were counseled for clinical exome testing, and one healthy subject was found to have a pathogenic *MLH1* variant Reference Genome Build GRCh37 (NM_000249.4): c.306G>T, p.(Glu102Asp).

## Discussion

This study from a large tertiary care oncology center in India involved 136 cases of various cancers, including colon, endometrial, ovarian, cervical, gastric, and a papillary thyroid carcinoma, along with a few healthy first-degree relatives of patients with Lynch-associated cancers. Of these, 53 cases were diagnosed with Lynch Syndrome, representing 38.39% of the total cohort, with colorectal cancer accounting for 71.7% (38 cases) and endometrial cancer for 28.3% (15 cases) (**Table 6**). Six cases had multiple malignancies at different times. Among the mutation-positive cases, 30 had pathogenic or likely pathogenic variants in the MLH1 gene, constituting 53.57% of this subgroup. The *MLH1* Reference Genome Build GRCh37 (NM_000249.4): c.306G>T, p.(Glu102Asp) variant was identified in 13 cases, accounting for 43.33% of MLH1 mutation cases and 23.21% of all Lynch syndrome cases. All carriers of this variant were from the Punjabi community in northwestern India. Three of these cases also had a strong family history of Lynch syndrome-related cancers, suggesting possible familial aggregation; however, no conclusions regarding penetrance can be drawn in the absence of segregation data. Sheth H et al. (13) reported the *MLH1* p.(Glu102Asp) variant as a potential founder mutation identified in Lynch syndrome patients of Indian ethnicity after haplotype analysis, comprising 25 Lynch syndrome carriers with the *MLH1* p.(Glu102Asp) variant and 100 healthy controls. The variant was estimated to have arisen in the population approximately 800 years ago. Nagabhushana P et al. have also reported *MLH1* p.(Glu102Asp) variant in a Lynch syndrome patient of Indian ancestry (14).

**Table 6:** Summary table of MMR-deficient tumors/MSI-high tumors with germline Lynch syndrome test result.

Total number of MMR-deficient tumor	MMR-deficient: 98 • Colon: 47 • Endometrium: 49 • Gastric: 1 • Cervix:1	MSI-high: 20 • Colon: 19 • Endometrium:1
Total number of germline confirmed-Lynch Syndrome	53 • Colon: 38 • Endometrium: 15	
Total number of cases with MMR-deficient tumor without confirmed germline Lynch syndrome	44	

The study highlighted several interesting findings, including that most frameshift variants result in premature protein truncation and loss of function. In this study, a case had *MSH6* (Reference Genome Build GRCh37) (NM_000179.3): c.4065_4066insGACT; p.(Leu1356Aspfs*4) frameshift variant, which has been categorized as a variant of uncertain significance in the ClinVar database (18). The variant caused protein truncation due to a frameshift insertion in the terminal exon, resulting in a protein that was only four amino acids shorter. The minimally shortened protein retains full functionality and may not disrupt it. Also the ClinVar database showed that the mutations after this region were mostly variant of uncertain clinical significance or benign functionally, further asserting our hypothesis that the minimally shortened protein may be functionally tolerated and unlikely to have a significant impact on protein function. However, no functional assay was performed for this variant due to logistic issues.

Another notable case showed loss of MLH1 and PMS2 expression in a grade 3 endometrioid endometrial carcinoma, while germline testing identified a frameshift pathogenic *MSH6* variant (NM_000179.3:c.3261del; p.(Phe1088Serfs*2), Reference Genome Build GRCh37). This represents a complex scenario involving multiple MMR defects. Similar paradoxical findings have been documented previously. In a large cohort of 703 individuals with a Lynch-like profile, Pan et al. reported 13 cases with IHC patterns discordant with their germline genetic findings. Notably, three cases showed loss of MLH1 and PMS2 on IHC despite carrying germline mutations in *MSH2* (two cases) or *MSH6* (one case) (19).

Several biologically plausible mechanisms can explain this discordance. First, the tumor may represent a phenocopy, arising through somatic *MLH1* promoter hypermethylation in a confirmed Lynch syndrome carrier. It is well recognized that not all tumors in Lynch syndrome carriers arise through the inherited MMR defect. Second, some truncating *MSH6* variants retain the epitope recognized by the IHC antibody, thereby preserving protein expression despite functional inactivation. This is particularly common for pathogenic *MSH6* variants in exons 4–5, which disrupt mismatch-binding but leave the antibody-binding region intact. Third, the tumor may have failed to acquire the “second hit” in *MSH6*, leaving MSH6 expression intact, while instead undergoing somatic *MLH1* inactivation, resulting in the observed MLH1/PMS2-loss phenotype (20).

In our cohort, MLH1/PMS2 loss was observed in 30 of 47 MMR-deficient colorectal cancers. *BRAF* p.(Val600Glu) was detected in 4 of 23 tested cases (17.4%), including 1 germline-confirmed Lynch syndrome case. MLH1 promoter hypermethylation was present in 4 of 10 cases, none of which were linked to Lynch syndrome, supporting its role as a reliable marker for sporadic tumors. *KRAS* mutations were found in 3 of 9 cases (33.3%), including 1 Lynch case.

Two cases from our cohort further highlight important diagnostic considerations when interpreting molecular alterations in the context of Lynch syndrome. First, we encountered a 72-year-old woman with a right-sided, poorly differentiated adenocarcinoma (pT4bN2, Stage IIIC) whose tumor demonstrated a *BRAF* V600E mutation while germline testing identified a pathogenic *PMS2* variant [NM_000535.7: c.2239A>T; p.(Arg747*), Reference Genome Build GRCh37]. Although *BRAF* V600E is classically associated with sporadic MLH1-deficient colorectal cancers, this case reinforces that *BRAF* mutations can occasionally coexist with Lynch syndrome. Therefore, the presence of a *BRAF* p.(Val600Glu) mutation alone should not be used to exclude germline testing, especially in patients with suggestive personal or family history or abnormal MMR IHC/MSI profiles.

Another illustrative case involved a patient with multiple primary malignancies over nearly two decades, including a rectosigmoid carcinoma (2003), transverse colon carcinoma (2021), and a small intestinal tumor (2022)—all of which exhibited MSI-high status. Tumor profiling (EntroGen assay) revealed a *KRAS* exon two mutation, while germline testing confirmed a pathogenic *MLH1* variant (NM_000249.4: c.306G>T; p.(Glu102Asp), Reference Genome Build GRCh37). This case emphasizes that *KRAS* mutations do not exclude Lynch syndrome, as they may occur in Lynch-associated tumors. In the presence of MSI-high status and a confirmed germline MMR gene pathogenic variant, such somatic mutations should be interpreted as part of the tumor’s broader mutational landscape rather than as criteria for dismissing hereditary cancer predisposition.

These findings are consistent with prior studies reporting rare *BRAF* or *KRAS* co-occurrence in Lynch syndrome. Collectively, these observations underscore that somatic driver mutations, such as *BRAF* or *KRAS*, may coexist with germline MMR pathogenic variants and, in isolation, should not determine eligibility for Lynch syndrome evaluation. Clinical context, tumor MMR phenotype, and germline testing remain essential for accurate diagnosis.

Current guideline-based algorithms use *BRAF* and/or *MLH1* methylation to triage *MLH1*-deficient tumors, reserving germline testing for marker-negative cases. Our data suggest that *BRAF* or *KRAS* status should not be used in isolation to exclude Lynch syndrome; germline testing remains appropriate in clinically suspicious cases. While broader testing could capture rare exceptions, practical considerations such as cost, laboratory capacity, and counseling availability must be taken into account. Overall, our results reinforce existing triage strategies while highlighting their limitations.

## Conclusion

This large single-center study delineates the genetic and clinicopathological spectrum of Lynch syndrome in a North Indian population, demonstrating that it accounts for a substantial proportion of colorectal and endometrial cancers and underscoring the importance of universal tumor screening and germline testing in MMR-deficient or MSI-high tumors. *MLH1* was the most frequently affected gene, with the recurrent *MLH1* Reference Genome Build GRCh37, NM_000249.4: c.306G>T; p.(Glu102Asp) variant representing nearly one-quarter of Lynch syndrome cases and occurring predominantly in individuals of Punjabi ancestry, strongly suggesting a regional founder mutation with implications for targeted and cost-effective genetic testing.

Strengths of the study include the large cohort size and the integration of clinical, histopathological, immunohistochemical, and genetic analyses.

The data in our study were derived from a single tertiary care referral center, which may introduce referral bias, as patients evaluated at specialized oncology centers often represent a clinically enriched population with a higher suspicion for hereditary cancer syndromes, such as Lynch syndrome. Therefore, the frequency and spectrum of mismatch repair deficiency and germline variants observed in this cohort may not fully represent the broader North Indian population. Future multicentric studies incorporating larger, more diverse cohorts from different regions of India should be conducted to validate these findings and provide a more representative understanding of the frequency of Lynch syndrome in the North Indian population. Other limitations include the lack of assessment for large genomic rearrangements and *EPCAM* deletions, which may have led to underestimation of *EPCAM*-mediated Lynch syndrome. Overall, these findings reinforce the need for population-specific data and an integrated diagnostic approach to enable accurate diagnosis, effective surveillance, and improved outcomes for patients and their families.

## Data Availability

The datasets presented in this study can be found in online repositories. The names of the repository/repositories and accession number(s) can be found in the article/**Supplementary Material**.
